# The promising immune checkpoint LAG-3 in cancer immunotherapy: from basic research to clinical application

**DOI:** 10.3389/fimmu.2022.956090

**Published:** 2022-07-26

**Authors:** Jin-Ling Huo, Ya-Tao Wang, Wen-Jia Fu, Nan Lu, Zhang-Suo Liu

**Affiliations:** ^1^ Traditional Chinese Medicine Integrated Department of Nephrology, the First Affiliated Hospital of Zhengzhou University, Research Institute of Nephrology, Zhengzhou University, Henan Province Research Center For Kidney Disease, Key Laboratory of Precision Diagnosis and Treatment for Chronic Kidney Disease in Henan Province, Zhengzhou, China; ^2^ Department of Orthopedics, First People’s Hospital of Shangqiu, Shangqiu, China; ^3^ School of Mechanical and Aerospace Engineering, Nanyang Technological University, Singapore, Singapore

**Keywords:** LAG-3, immune checkpoint, cancer immunotherapy, tumor microenvironment, T cell

## Abstract

LAG-3, a type of immune checkpoint receptor protein belonging to the immunoglobulin superfamily, is confirmed to be expressed on activated immune cells, mainly including activated T cells. LAG-3 can negatively regulate the function of T cells, exerting important effects on maintaining the homeostasis of the immune system under normal physiological conditions and promoting tumor cells immune escape in the tumor microenvironment. Given its important biological roles, LAG-3 has been regarded as a promising target for cancer immunotherapy. To date, many LAG-3 inhibitors have been reported, which can be divided into monoclonal antibody, double antibody, and small molecule drug, some of which have entered the clinical research stage. LAG-3 inhibitors can negatively regulate and suppress T cell proliferation and activation through combination with MHC II ligand. Besides, LAG-3 inhibitors can also affect T cell function *via* binding to Galectin-3 and LSECtin. In addition, LAG-3 inhibitors can prevent the FGL1-LAG-3 interaction, thereby enhancing the human body’s antitumor immune effect. In this review, we will describe the function of LAG-3 and summarize the latest LAG-3 inhibitors in the clinic for cancer therapy.

## Introduction

A large amount of evidence from literature has manifested that tumor cells can effectively avoid being recognized and killed by the immune system through immune checkpoint receptor proteins, suggesting that blocking immune checkpoint receptors is a new immunotherapy for human cancers (1, 2). The most well-studied immune checkpoint receptors mainly include programmed cell death 1/programmed cell death 1 ligand (PD-1/PD-L1) and cytotoxic T lymphocyte-associated antigen-4 (CTLA-4) (3–6). However, due to the treatment tolerance, low response, or significant increase in toxicity of previously discovered antibody drugs targeting PD-1/PD-L1 or CTLA-4 (7–9), it is very necessary to investigate new targets against immune checkpoint receptor proteins.

Lymphocyte activation gene-3 (LAG-3, also named CD223 or FDC protein), is a new class of immune checkpoint receptors, which was first isolated and reported by French immunologist Frédéric Triebel and colleagues in 1990 (10). LAG-3, as a key member of the immunoglobulin superfamily (IgSF) locating on human chromosome 12, is a type I transmembrane protein containing 498 amino acids, consisting of transmembrane region, the extracellular region, and cytoplasmic region (11). The expression level of LAG-3 is closely related to the prognosis of human tumors. High level of LAG-3 in kidney renal clear cell carcinoma, non-small cell lung cancer (NSCLC), primary central nervous system lymphoma (PCNSL), hepatocellular carcinoma (HCC) and muscle invasive bladder cancer (MIBC) indicates a poor prognosis, whereas in gastric carcinoma and melanoma predicts a better prognosis (12). LAG-3 is detected to be expressed on the surface of effector T cells and regulatory T cells (Tregs) that participate in the regulation of T lymphocytes and antigen-presenting cells (APCs) signaling pathways and play a crucial part in the adaptive immune response (13). Consistent with CTLA-4 and PD-1/PD-L1, LAG-3 is induced on CD8+ and CD4+ T cells upon persistent antigenic stimulation, rather than expressed on naive T cells (14). Since the inhibitory function of LAG-3 is closely associated with its expression level on the activated immune cells, the blockage and inhibition of LAG-3 expression through antibody drugs or small molecule inhibitors are critical. Prolonged infection with viruses, fungus, and bacteria results in sustained exposure to antigens, leading to high levels of persistent expression of LAG-3 and other inhibitory co-receptors on CD8+ and CD4+ T cells (15). These T cells lose powerful effector functions, known as exhausted T cells, resulting in decreased tumor lethality and response rate, and upregulation of Treg immunosuppressive function (15). Studies have shown that blockage or inhibition of LAG-3 can allow T cells to regain cytotoxic activity and reduce the function of regulating T cells to suppress immune responses, thereby enhancing the killing effect on tumors (16, 17). It was observed that simultaneous blockage of LAG-3 activity and anti-PD-1 or PD-L1 in tumor cells has dual inhibitory effects, including inhibiting Treg activity, promoting dendritic cells (DCs) maturation, and rescuing dysfunctional CD4+/CD8+ T cells (18–20). LAG-3 has been regarded as an indicator of tumor prognosis and become a novel tumor immunotherapy target beyond PD-1/PD-L1 and CTLA-4. Herein, we aim to describe the structure and the known ligands of LAG-3 and summarize the immune-regulatory effects on active T cells in tumor microenvironment, as well as the LAG-3 inhibitors which have been evaluated in the clinic.

## LAG-3 structure and ligands

**Figure 1:**
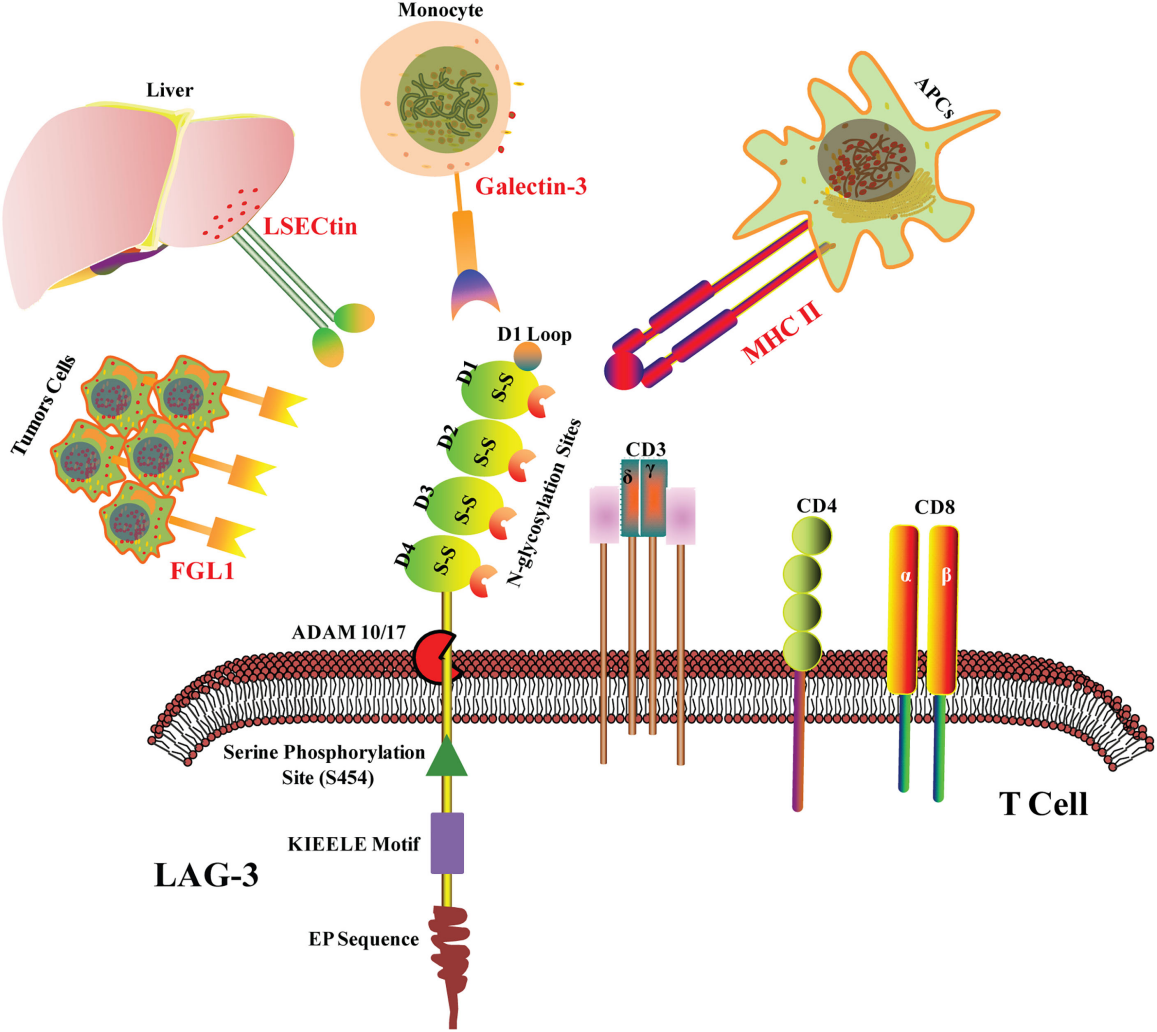
LAG-3 structure and ligands. LAG-3 consists of extracellular region, transmembrane region and cytoplasmic region. The extracellular domain is composed of four IgSF domains, namely D1, D2, D3 and D4. The D1 domain contains a loop domain rich in proline and an in-chain disulfide bond. In the transmembrane- cytoplasmic part, LAG-3 breaks away from the cell membrane under the action of metalloproteinases ADAM10/17. The cytoplasmic region of LAG-3 consists of three parts: the serine phosphorylation site S454, the highly conserved “KIEELE” motif and the glutamate-proline dipeptide repeat motif (EP sequence). MHC II, Galectin-3, LSECtin and FGL1 are the confirmed ligands of LAG-3 in tumor microenvironment.

LAG3 co-localizates with CD4, CD8, and CD3 molecules within lipid rafts. The structure of LAG-3 is essentially different from that of CD3 and CD8, whereas it is highly homologous to CD4. LAG-3 consists of three parts: transmembrane region, extracellular region, and cytoplasmic region. In the transmembrane-cytoplasmic part, LAG-3 breaks away from the cell membrane under the action of metalloproteinases ADAM10/17, which can regulate the function of LAG-3. The extracellular domain is responsible for binding to the ligands and consists of four IgSF domains, namely D1, D2, D3, and D4. The D1 domain contains a loop domain rich in proline (~30) and an in-chain disulfide bond, which is species-specific and is known as the V immunoglobulin superfamily. However, D2, D3, and D4 belong to the C2 family. The cytoplasmic region of LAG-3 consists of three parts: the serine phosphorylation site S454 (substrates for protein kinase C, PKC), the highly conserved “KIEELE” motif, and the glutamate-proline dipeptide repeat motif (EP sequence) (**Figure 1**) (21, 22). Importantly, KIEELE mutant resulted in complete loss of LAG-3 function, which proved that the “KIEELE” motif was crucial to the function of LAG-3 (23).

LAG-3 was detected to be mainly expressed on the surface of activated T cells (CD8+ T cells and CD4+ T cells), naturalkiller cells (NK cells), B cells, and DCs under physiological conditions, and negatively regulate T cell function. Researchers also found a small number of LAG-3+ lymphocytes in inflammatory lymphoid tissues, such as tonsils or lymph nodes (24). In pathological state, LAG-3 was reported to be highly expressed on the surface of tumor-infiltrating lymphocytes (TILs), the expression level of which was positively correlated with the occurrence and development of human tumors, such as non-small cell lung cancer (NSCLC) and hepatocellular carcinoma (HCC) (25–27). LAG-3 negatively regulates the function of T cells and plays significant roles in maintaining the homeostasis of immune system under normal physiological conditions and promoting tumor cells immune escape in the tumor microenvironment, indicating a promising target for tumor immunotherapy.

It has been reported four ligands of LAG-3 in tumor microenvironment mainly including galactose lectin-3 (Galectin-3), major histocompatibility complex II (MHC II), fibrinogen-like protein 1 (FGL1), and hepatic sinusoid endothelial cell lectin (LSECtin) (**Figure 1**). MHC II is the main ligand of LAG-3 (28). Due to the high homology of LAG3 and CD4, MHC II is the common ligand of LAG3 and CD4. However, LGA3 and MHC II shows 100 times higher in binding affinity than CD4, suggesting that CD4 and LAG3 may competitively bind to MHC II, thereby negatively regulating the function of CD4 (29, 30). Studies have shown that although LAG3 mutants unable to bind MHC II exhibit reduced inhibitory function (21), tail mutations in the intracellular domain of LAG3 lead to loss of inhibitory effect, further suggesting that the intracellular domain is critical for inhibiting signal transduction (23). It is possible that LAG3 acts not primarily by interfering with the interaction of MHC II and CD4, but rather by transmitting inhibitory signals *via* the cytoplasmic domain (23), although the exact character of this signal is unclear.

Galectin-3, another ligand of LAG-3, is a 31-kDa soluble lectin (**Figure 1**). Studies have shown that LAG3 is highly glycosylated and can interact with Galectin-3, which regulates T cell responses *via* several mechanisms (28). *In vitro* experiments showed that LAG3 played important roles in Galectin-3-mediated inhibition of IFN-γ secretion by CD8+ T cells (31). Furthermore, Galectin-3 expressed by a variety of cells in the tumor microenvironment instead of the tumor itself may interact with LAG3 on tumor-specific CD8+ T cells, thus resulting in the modulation of anti-tumor immune responses (32). LSECtin, a potential ligand of LAG-3, belongs to the C-type lectin receptor superfamily and is mainly expressed in liver (33). LSECtin has also been found in human melanoma tissues. The interaction between LSECtin and LAG-3 promotes tumor growth through suppression of anti-tumor T cell response in melanoma cells (34).

Jun Wang et al. found that FGL1 is an immune inhibitory ligand of LAG-3 independent of MHC II (**Figure 1**). LAG-3 binds with FGL1 through the domains of D1 and D2. The interaction between FGL1 and LAG-3 mutually promotes tumor immune escape through inhibiting the activation of antigen-specific T cell (35). Notably, a recent study has revealed that the binding of LAG-3 to MHC II but not to FGL1 mediated the suppression of T cells (36). Of course, other LAG3 ligands have not yet been discovered. In addition, a study has shown that LAG3 binds preformed fibrils of α-synuclein in the central nervous system, thereby promoting the pathogenesis of Parkinson’s disease in a mouse model (37), suggesting that LAG3 may also have functions outside the immune system.

## LAG-3 immunological functions

**Figure 2:**
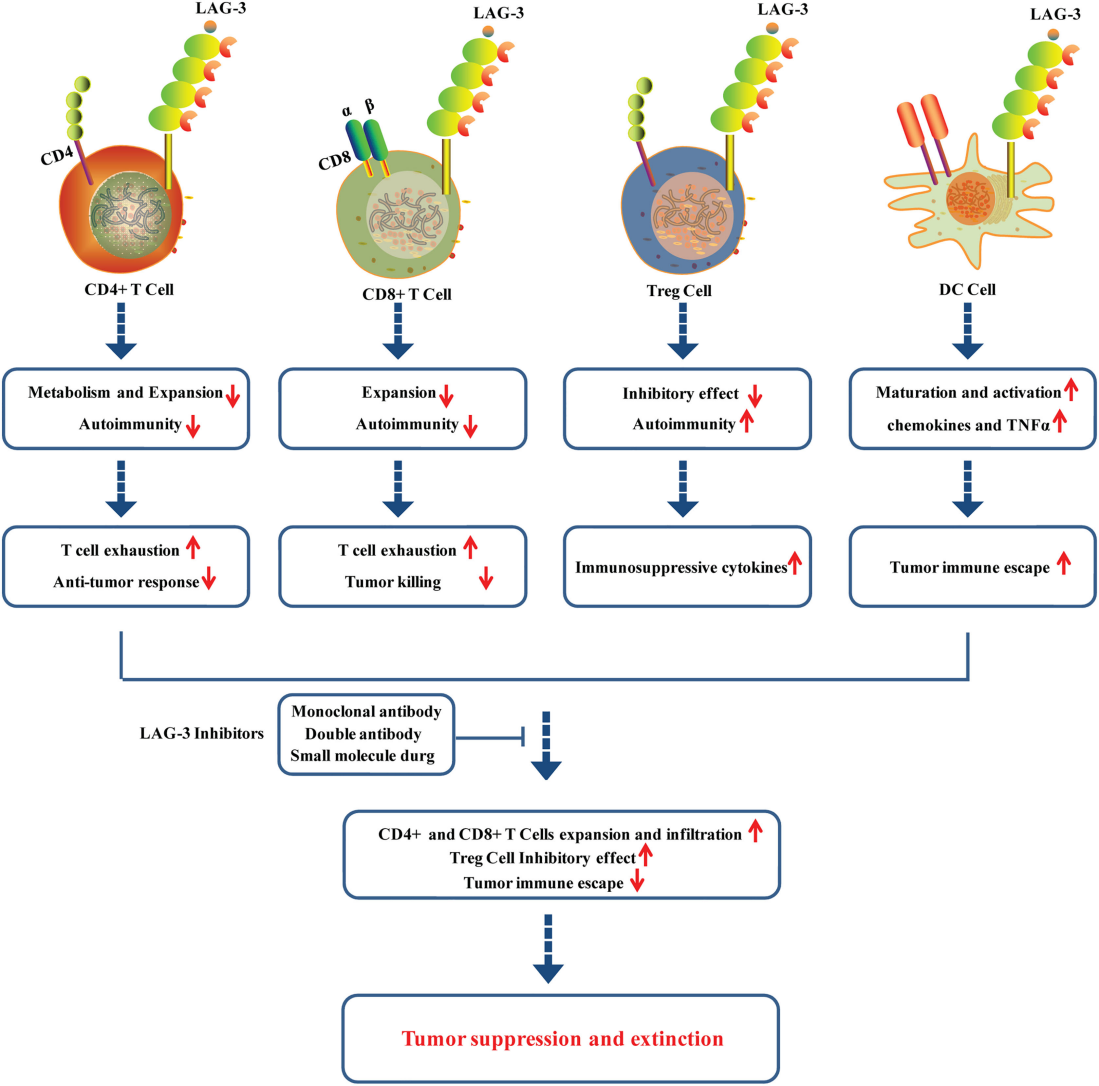
Roles of LAG-3 in CD+4 cells, CD8+ cells, Treg cells and DC cells in tumor microenvironment.

LAG-3 interacts with its ligands to regulate the function of T cells. The interaction between MHC II and LAG-3 can down-regulate the cytokine secretion level and proliferation ability of CD4+ T cells (**Figure 2**). The anti-LAG-3 antibody can restore CD4+ T cells activity. Nevertheless, the specific regulatory mechanism remains unknown (38, 39). It is worth noting that LAG-3 selectively binds to antigen peptide-MHC II (pMHC II), thus inhibiting pMHC II-responsive CD4+ T cells (40, 41). LAG-3 was found to negatively regulate the mitochondrial activity in naive CD4+ T cells, restricting the normal metabolism and expansion of naive CD4+ T cells and leading to T cell exhaustion and anti-tumor response (42). In addition, LAG-3 was also observed to be upregulated in CD8+ T cells stimulated with tumor antigens (**Figure 2**) (43). CD8+ T cells in LAG-3-deficient mice exhibited significantly higher activity than that in normal mice, suggesting that LAG-3 has an inhibitory effect on CD8+ T cells. LAG-3 has been demonstrated to directly inhibit CD8+ T cells *via* signal transduction, independent of the role of MHC II and CD4+ T cells (44, 45). LAG-3 can also enhance the function of regulatory T cells (Treg cells) (**Figure 2**). Treg cells play a negative role in immune regulation and can down-regulate T cell activity. Common types of Treg cells include natural regulatory T cells (nTreg cells) and inducible regulatory T cells (iTreg cells). LAG-3 can positively induce Treg cells activation and stimulate their immunosuppressive function (46–48). LAG-3 may synergize with other inhibitory molecules (PD-1, CTLA-4) to improve the inhibitory activity of Treg cells, leading to APC-induced immune tolerance (49).

LAG-3 also plays immune adjuvant roles and participates in the tumor immune escape. LAG-3 can induce the maturation and activation of DC cells through regulation of intracellular protein phosphorylation and promotion of the chemokines and tumor necrosis factor α (TNFα) production (**Figure 2**) (50). LAG-3 highly expressed on the TILs interacts with ligands located on the surface of tumor cells to cause T cell dysfunction or even exhaustion, promoting tumor immune escape, the phenomenon of which is particularly evident in CD8+ T cells (25, 51, 52). Moreover, it is confirmed that LAG-3 displayed potential roles in activation of NK cells, although its underling mechanisms remains to be further studied.

## Roles of blocking LAG-3 in the tumor microenvironment

LAG-3 is confirmed to be highly expressed on TILs of various solid tumors, including colon cancer, NSCLC, head and neck cell cancer, and pancreatic cancer (18, 53–55). LAG-3 has been revealed to play a vital role in regulating T cell activation, proliferation, homeostasis, and T cell-depleted immune microenvironments. LAG-3 was also found to be co-expressed with PD-1 in the tumor microenvironment. LAG-3 and PD-1 induced T cell function inhibition through different signaling pathways, which may synergistically lead to exhaustion of T cells. Studies have shown that co-blockade of PD1 and LAG3 expressed on CD8+ and CD4+ TILs exhibited enhanced antitumor responses in some preclinical mouse models of ovarian cancer, colon adenocarcinoma, and melanoma (52, 56, 57). LAG3 expressed on iTreg cells induces the production of TGF-β1 and IL-10, which contributes to tumor immune escape. The blocking LAG3 antibodies can reduce the inhibitory effect of Tregs, resulting in the restoration of CD8+ TIL activity (57, 58) (**Figure 2**).

Studies have shown that inhibition of LAG-3 can allow T cells to regain cytotoxic activity and reduce the function of regulating T cells to suppress immune responses, enhancing the killing effect on tumors (59) (**Figure 2**). Blocking LAG-3 activity and anti-PD-1 or PD-L1 in tumor cells has dual inhibitory effects, including inhibiting Treg activity, promoting DC maturation, and rescuing dysfunctional CD4+/CD8+ T cells (60). LAG-3 has become a novel tumor immunotherapy target beyond CTLA-4 and PD-1/PD-L1. The overall drug types of LAG-3 inhibitors can be divided into monoclonal antibody, double antibody, and small molecule drug, some of which have entered the clinical research stage. More than 80 clinical trials are underway globally to evaluate the drug candidates targeting LAG-3.

LAG-3 inhibitors can directly bind LAG-3 molecules or their ligands, blocking the interaction between ligands and LAG-3, and downregulating the inhibitory efficacy of LAG-3 toward the immune system. LAG-3 antibodies not only restore T cell function, but also inhibit Treg cells activity. In previous studies, antibodies against PD-1 can only activate T cells, but cannot inhibit the activity of Treg cells (61–63). Taken together, LAG-3 inhibitors may have a better therapeutic effect, further demonstrating a novel tumor immunotherapy target of LAG-3 beyond PD-1/PD-L1 and CTLA-4.

## Clinical development of LAG-3 targeted cancer immunotherapy

As a promising target for cancer immunotherapy, LAG3 has been hotly pursued by academia and pharmaceutical companies. In the past, significant progress has been made in the discovery of many LAG3 modulators and some of them are currently in the clinic as anticancer drugs, which are summarized in **Table 1**, involving LAG3-targeted cancer immunotherapy that are either completed, ongoing, or recruiting participants (ClinicalTrials.gov). Eftilagimod alpha, developed by Immutep S.A.S. as the initial first-in-class LAG3 modulator, could activate APCs *via* interacting with canonical ligand (MHC class II), which has been also found to enhance Treg immunosuppression, stimulate the proliferation of DCs, and ameliorate antigen crosspresentation to CD8+ T cells (64). Three clinical trials for Eftilagimod alpha have been completed, and three others are recruiting participants as shown in **Table 1**. In addition, 10 different LAG3-specific monoclonal antibodies and six bispecific antibodies are currently under investigation at various clinical stages for the treatment of cancer (**Table 1**). As the first anti-LAG3 human IgG4 monoclonal antibody and novel immune checkpoint inhibitor, relatlimab, discovered by Bristol-Myers Squibb, is currently undergoing 46 different clinical trials for cancer therapy (65). As the first commercially developed anti-LAG-3 antibody, relatlimab entered the clinical trials in 2013 (66). However, due to the limited efficacy of relatlimab alone, it is generally used in combination with other checkpoint inhibitors, including CTLA-4 inhibitors (ipilimumab) or PD-1 inhibitors (nivolumab), to synergistically improve the efficacy (39). Encouragingly, relatlimab in combination with the PD-1 inhibitor nivolumab received FDA approval in March 2022 as the first approved monoclonal antibody to treat unresectable or metastatic melanoma (67).

**Table 1:** LAG3-modulating candidates^a^.

Drug	Phase	ClinicalTrials.gov ID	Indications	Status
Nivolumab/Relatlimab	Phase 1	NCT04658147	Hepatocellular Carcinoma	Recruiting
	Phase 1	NCT02966548	Cancer	Active, not recruiting
	Phase 1	NCT03335540	Advanced Cancer	Active, not recruiting
	Phase 1	NCT03044613	Gastric Cancer	Active, not recruiting
	Phase 1/2	NCT03459222	Advanced Cancer	Recruiting
	Phase 1/2	NCT02061761	Hematologic Neoplasms	Recruiting
	Phase 1/2	NCT03310619	Lymphoma	Recruiting
	Phase 1/2	NCT02488759	Various Advanced Cancer	Active, not recruiting
	Phase 1/2	NCT03610711	Gastroesophageal Cancer Immune Checkpoint Inhibition	Recruiting
	Phase 1/2	NCT04611126	Metastatic Ovarian Cancer	Recruiting
	Phase 1/2	NCT05134948	Advanced Solid Tumors	Recruiting
	Phase 1/2	NCT03978611	Melanoma	Recruiting
	Phase 1/2	NCT05337137	Carcinoma, Hepatocellular	Recruiting
	Phase 1/2	NCT05255601	Lymphoma, Non-Hodgkin Hodgkin Disease	Not yet recruiting
	Phase 1/2	NCT04150965	Multiple Myeloma	Recruiting
	Phase 2	NCT04552223	Metastatic Uveal Melanoma	Recruiting
	Phase 2	NCT04095208	Soft Tissue Sarcoma Adult Advanced Cancer	Recruiting
	Phase 2	NCT03623854	Chordoma	Recruiting
	Phase 2	NCT03743766	Melanoma	Recruiting
	Phase 2	NCT04080804	Head and Neck Squamous Cell Carcinoma	Recruiting
	Phase 2	NCT04913922	Acute Myeloid Leukemia	Recruiting
	Phase 2	NCT05002569	Melanoma	Recruiting
	Phase 2	NCT04112498	Cancer	Active, not recruiting
	Phase 2	NCT03607890	Refractory MSI-H Solid Tumors Prior of PD-(L) 1 Therapy MSI-H Tumors	Recruiting
	Phase 2	NCT03642067	Microsatellite Stable (MSS) Colorectal Adenocarcinomas Colorectal Adenocarcinoma	Recruiting
	Phase 2	NCT03724968	Melanoma	Terminated
	Phase 2	NCT03704077	Gastric Cancer	Withdrawn
	Phase 2	NCT02750514	Advanced Cancer	Terminated
	Phase 2	NCT04567615	Hepatocellular Carcinoma	Recruiting
	Phase 2	NCT03521830	Basal Cell Carcinoma	Recruiting
	Phase 2	NCT04326257	Squamous Cell Carcinoma of the Head and Neck	Recruiting
	Phase 2	NCT04623775	Non-small Cell Lunch Cancer	Recruiting
	Phase 2	NCT05347212	Carcinomas	Not yet recruiting
	Phase 2	NCT04205552	NSCLC Stage I/II/IIIA	Recruiting
	Phase 2	NCT03867799	Metastatic Colorectal Cancer	Active, not recruiting
	Phase 2	NCT05148546	Renal Cell Carcinoma	Recruiting
	Phase 2	NCT01968109	Neoplasms by Site	Active, not recruiting
	Phase 2	NCT03662659	Gastric Cancer	Active, not recruiting
	Phase 2	NCT02519322	Stage IIIB-IV melanoma	Active, not recruiting
	Phase 2	NCT04062656	Gastric Cancer	Recruiting
	Phase 2	NCT02996110	Advanced Cancer	Active, not recruiting
	Phase 2	NCT02935634	Advanced Gastric Cancer	Active, not recruiting
	Phase 2	NCT02465060	solid tumors or lymphomas	Recruiting
	Phase 2/3	NCT03470922	Melanoma	Active, not recruiting
	Phase 3	NCT05328908	Colorectal Neoplasms	Recruiting
	Not Applicable	NCT04866810	Melanoma	Recruiting
Tebotelimab	Phase 1	NCT03219268	HER2-positive Advanced Solid Tumors	Active, not recruiting
	Phase 2	NCT04634825	Head and Neck Cancer, Neoplasms, Squamous Cell Carcinoma	Recruiting
	Phase 2/3	NCT04082364	Gastric Cancer	Active, not recruiting
Chlorogenic acid	Phase 1	NCT02728349	Glioblastoma	Completed
	Phase 1	NCT02245204	Advanced Cancer	Completed
	Phase 1	NCT02136342	Advanced Cancer	Terminated
	Phase 1/2	NCT03751592	Advanced Lung Cancer	Unknown
RO-7247669	Phase 1	NCT04140500	Solid Tumors	Recruiting
	Phase 1/2	NCT04524871	Advanced Liver Cancers	Recruiting
	Phase 1/2	NCT05116202	Melanoma	Recruiting
	Phase 2	NCT04785820	Advanced or Metastatic Esophageal Squamous Cell Carcinoma	Recruiting
Favezelimab	Phase 1	NCT02720068	Neoplasms	Active, not recruiting
	Phase 1/2	NCT04938817	Small Cell Lung Carcinoma	Recruiting
	Phase 1/2	NCT04626479	Carcinoma, Renal Cell	Recruiting
	Phase 1/2	NCT05342636	Esophageal Squamous Cell Carcinoma (ESCC)	Not yet recruiting
	Phase 1/2	NCT03598608	Hodgkin Disease Lymphoma	Recruiting
	Phase 1/2	NCT04626518	Carcinoma, Renal Cell	Recruiting
	Phase 2	NCT04895722	Colorectal Cancer	Recruiting
	Phase 2	NCT03516981	Advanced Non-Small Cell Lung Cancer	Active, not recruiting
	Phase 3	NCT05064059	Colorectal Cancer	Recruiting
INCAGN-2385	Phase 1	NCT03538028	Advanced Malignancies	Completed
	Phase 1/2	NCT04370704	Melanoma	Recruiting
	Phase 2	NCT05287113	Head and Neck Cancer	Not yet recruiting
	Phase 2	NCT04586244	Urothelial Carcinoma	Recruiting
IBI-110	Phase 1	NCT04085185	Advanced Malignancies	Recruiting
	Phase 1	NCT05039658	DLBCL	Not yet recruiting
	Phase 2	NCT05026593	SCLC	Recruiting
	Phase 2	NCT05088967	Non-small Cell Lung Cancer	Recruiting
Eftilagimod alpha	Phase 1	NCT02676869	Stage III/IV Melanoma	Completed
	Phase 1	NCT04252768	Metastatic Breast Cancer	Not yet recruiting
	Phase 1	NCT03600090	Solid Tumor, Adult	Completed
	Phase 2	NCT03625323	Non-small cell lung carcinoma, head and neck carcinoma	Active, not recruiting
	Phase 2	NCT04811027	HNSCC	Recruiting
	Phase 2	NCT02614833	Adenocarcinoma Breast Stage IV	Completed
Sym-022	Phase I	NCT03489369	Metastatic Cancer, Solid Tumor Lymphoma	Completed
	Phase I	NCT04641871	Metastatic Cancer Solid Tumor	Recruiting
	Phase 1	NCT03311412	Metastatic Cancer, Solid Tumor Lymphoma	Completed
	Phase I	NCT04414150	Malignant Tumors	Unknown
	Phase 2	NCT05208177	Advanced Solid Tumor	Not yet recruiting
LBL-007	Phase 1	NCT04640545	Advanced Melanoma	Recruiting
	Phase 1/2	NCT05102006	Advanced Solid Tumor	Recruiting
ABL-501	Phase I	NCT05101109	Advanced Solid Tumor	Recruiting
Anti-LAG3 antibody	Phase 1	NCT02658981	Glioblastoma	Active, not recruiting
HLX 26	Phase I	NCT05078593	Solid Tumor	Recruiting
IBI-323	Phase I	NCT04916119	Advanced Malignancies	Recruiting
Ieramilimab	Phase 1/2	NCT02460224	Advanced Solid Tumors	Completed
FS 118	Phase 1/2	NCT03440437	Advanced Cancer	Recruiting
EMB-02	Phase 1/2	NCT04618393	Advanced Solid Tumors	Recruiting
Fianlimab	Phase 3	NCT05352672	Melanoma	Not yet recruiting

a The data were from https://www.clinicaltrials.gov/#opennewwindow.

## Discussion

Since its discovery in 1990, LAG3 has gained widespread interest and been regarded as a promising target for cancer immunotherapy. LAG-3 plays an important immunoregulatory role in a variety of human tumors, and blocking LAG-3 can enhance the proliferation of TILs and the secretion of cytokines, and enhance anti-tumor immunity. Many LAG3 inhibitors have been discovered and are currently in the clinic. Single anti-LAG3 therapy was demonstrated to be modest benefit, supporting a potential combination approach with other inhibitory receptors. LAG-3 inhibitors, together with CTLA-4 or PD-1/PD-L1 inhibitors, have been extensively explored in the different clinical trials for cancer therapy, which can not only avoid drug tolerance but also improve the clinical efficacy of LAG-3 inhibitors. No evidence reveals the feasibility of the combination between LAG-3 inhibitors and other immune checkpoint inhibitors. So far, the regulatory mechanism of LAG-3 has not been fully explored and the clinical efficacy of its inhibitors is uncertain. Based on the current clinical data, the early therapeutic effect of LAG-3 monoclonal antibody is not satisfactory. According to the phase I clinical data of LAG-3 monoclonal antibody MK-4280 published by Merck, the objective response rate (ORR) among 18 patients with solid tumors that failed other treatments was only 6%, and the disease control rate is only 17%. Therefore, it is mainly to explore the combination strategy, especially the combination of LAG-3 and PD-1. The bi-functional monoclonal antibody is worthy of attention and exploration. There are only a few interim reports of the combination therapies targeting LAG-3 and PD-1. Evidence revealed that the combination exerted better tolerance and higher ORR, extended progression-free survival, as well as a lower risk of death (13). The exact efficacy of anti-LAG-3 antibodies as monotherapy and the additive effects of anti-LAG-3 antibodies in the combination therapy targeting PD-1 and LAG-3 need to be further explored.

Inevitably, there are still many questions that remain to be resolved regarding the understanding of LAG3 biology, the exact signaling pathway and the potential ligands, as well as the mechanism underlying synergistic effect with other immune checkpoint molecules, although the development of LAG-3 inhibitors is in full swing. If these problems could be solved, the research on LAG-3 and its related drugs will make significant progress for cancer therapy.

## Author contributions

J-LH and NL conceptualized the ideas. Y-TW, W-JF, J-LH, and NL drafted the original manuscript. Z-SL revised the manuscript. All the authors approved the final version of the manuscript.

## Funding

This work was financially supported by the General Program of the National Natural Science Foundation of China General Project (No. 81970633) and National Natural Science Foundation of China Joint project (NO. U21A20348).

## Conflict of interest

The authors declare that the research was conducted in the absence of any commercial or financial relationships that could be construed as a potential conflict of interest.

## Publisher’s note

All claims expressed in this article are solely those of the authors and do not necessarily represent those of their affiliated organizations, or those of the publisher, the editors and the reviewers. Any product that may be evaluated in this article, or claim that may be made by its manufacturer, is not guaranteed or endorsed by the publisher.
